# Sodium Alginate- and Cationic Cellulose-Functionalized Polycaprolactone Nanofibers for In Vitro and Antibacterial Applications

**DOI:** 10.3390/molecules28217305

**Published:** 2023-10-27

**Authors:** Emad Tolba, Ahmed Salama, Ahmed K. Saleh, Iriczalli Cruz-Maya, Vincenzo Guarino

**Affiliations:** 1 Polymers and Pigments Department, National Research Centre, 33 El-Buhouth St., Dokki, Cairo 12622, Egypt; emad_nrc@yahoo.com; 2 Cellulose & Paper Department, National Research Centre, 33 El Bohouth St., Dokki, Giza 12622, Egypt; asrk_saleh@yahoo.com; 3 Institute of Polymers, Composite and Biomaterials, National Research Council of Italy, Mostra d’Oltremare, V.le J.F. Kennedy 54, 80125 Naples, Italy

**Keywords:** cationic cellulose, electrospun fibers, antimicrobial properties, wound healing

## Abstract

The use of polyelectrolytes is emerging as a fascinating strategy for the functionalization of biomedical membranes, due to their ability to enhance biological responses using the interaction effect of charged groups on multiple interface properties. Herein, two different polyelectrolytes were used to improve the antibacterial properties of polycaprolactone (PCL) nanofibers fabricated via electrospinning. First, a new cationic cellulose derivative, cellulose-bearing imidazolium tosylate (CIMD), was prepared via the nucleophilic substitution of the tosyl group using 1-methylimidazole, as confirmed by NMR analyses, and loaded into the PCL nanofibers. Secondly, sodium alginate (SA) was used to uniformly coat the fibers’ surface via self-assembly, as remarked through SEM-EDX analyses. Polyelectrolyte interactions between the CIMD and the SA, initially detected using a FTIR analysis, were confirmed via Z potential measurements: the formation of a CMID/SA complex promoted a substantial charge neutralization of the fibers’ surfaces with effects on the physical properties of the membrane in terms of water adsorption and in vitro degradation. Moreover, the presence of SA contributed to the in vitro response of human mesenchymal stem cells (hMSCs), as confirmed by a significant increase in the cells’ viability after 7 days in the case of the PCL/CMID/SA complex with respect to the PCL and PCL/CMID membranes. Contrariwise, SA did not nullify the antibacterial effect of CMID, as confirmed by the comparable resistance exhibited by *S. mutans*, *S. aureus,* and *E. coli* to the PCL/CIMD and PCL/CIMD/SA membranes. All the reported results corroborate the idea that the CIMD/SA functionalization of PCL nanofibers has a great potential for the fabrication of efficient antimicrobial membranes for wound healing.

## 1. Introduction

Skin injuries, particularly chronic wounds, are a global healthcare problem that can be treated with the use of wound-dressing materials that speed-up the healing process [1,2,3]. As a result of their peculiar structural properties, electrospun polymeric nanofibrous nonwovens can be used as functional wound-dressing materials. By using electrospun nonwoven nanofibers, wound contamination and additional physical damage can be prevented. The nanofibrous membrane can act as a template structure for supporting skin cells during the repair process in order to minimize the formed tissue scar. Their nanostructured network allows the creation of a unique, interconnected, and porous 3D architecture able to facilitate the extraction of extra bodily fluids (i.e., exudate) from the wound area. The purpose of this is to prevent infection and maintain a moist environment, reducing wounds’ healing time without forming scabs [4]. Several studies have been carried out to incorporate antibacterial agents, such as metal nanoparticles, active polysaccharides, and natural extracts, into nanofibrous scaffolds [5,6,7].

The production of polymeric nanofibers—i.e., fibers with diameters measuring less than 1 µm—has been carried out using a variety of processes, including phase-separation, self-assembly, template synthesis, solution blow spinning, and force spinning [8,9,10,11]. Among them, electrospinning provides a simple and highly versatile approach for fabricating ultrafine polymer fiber arrays with tunable physical and chemical properties [12,13]. One of the most commonly electrospun polymers is polycaprolactone (PCL), due to its high biocompatibility and excellent mechanical properties, also under in vitro conditions. Moreover, PCL is one of the first synthetic polyesters approved for biomedical applications by the U.S. Food and Drug Administration (FDA) [14,15,16]. However, its application in wound healing is still strongly limited, generally requiring the incorporation of antimicrobial reagents to promote antimicrobial properties.

It is well known that the progression of the wound healing process involves several coexisting phases—inflammation, proliferation, and remodeling— to achieve a successful wound repair [17,18]. The inflammatory phase begins immediately after a tissue injury, due to the disruption of blood vessels and the releasing of coagulation factors. During the inflammatory phase, different types of leukocytes can migrate from neighboring blood vessels to clean-up the injured area. This can contribute to the generation of inflammatory mediators, acting as stimulating factors for the next phase, namely, the proliferative phase. During this step, the closure of the trauma occurs through the formation of a protein matrix, mainly made of collagen. The final step is called remodeling and is marked by the extracellular matrix (ECM)’s maturation in achieving the maximum tensile strength, and it is associated with scar-tissue formation. Among the recent advances in wound-healing management is the fabrication of synthetic skin-tissue-like materials which could replace damaged skin tissue and accelerate the healing process [19]. For the use of these materials, a certain mechanical stability of the wound related to specific environmental conditions—for instance, wound moisture—is required to recreate the mechanical strength conditions able to protect and support various skin cells’ activities during the repair process up until the physiological closure of the wound. Moreover, biodegradability is required to gradually provide space to the forming tissue and create a microenvironment able to support the healing action, protect tissue from mechanical trauma, and provide a physical barrier against bacterial invasion.

Furthermore, recent studies have confirmed that functional wound-dressing materials are highly desired to guide the healing process and fight against bacterial invasion [20]. In this view, the preparation of novel biopolymers with antimicrobial properties has been investigated in numerous areas of application, especially in wound healing. The high hydroxyl content of cellulose makes it an ideal material for making multifunctional products [21]. Cellulose is a linear homopolysaccharide formed from D-anhydroglucopyranose units linked with glycosidic bonds. The three hydroxyl groups form strong hydrogen bonds within and between the molecules, resulting in crystalline and amorphous fibrils. Many trials have focused on the functionalization of cellulose for the preparation of new derivatives with promising applications [22]. The preparation of cellulose derivatives with tetrabutylammonium moieties has been carried out through the acidification of carboxymethyl cellulose, followed by acid–base neutralization using tetrabutylammonium hydroxide. According to their antibacterial behavior, the new carboxymethyl cellulose–tetrabutylammonium derivatives are extremely active against bacteria depending on cationization level and bacteria type [23].

In this work, the fabrication of PCL nanofibers functionalized using cationic cellulose (CIMD) and sodium alginate (SA) is proposed as a means to investigate the effect of the interaction between CIMD and SA on the response of in vitro cells and bacteria. The CIMD was incorporated into the PCL nanofibers during the electrospinning process, while the SA was integrated via self-assembly as a fiber coating.

## 2. Results and Discussion

### 2.1. Subsection

**Figure 1:**
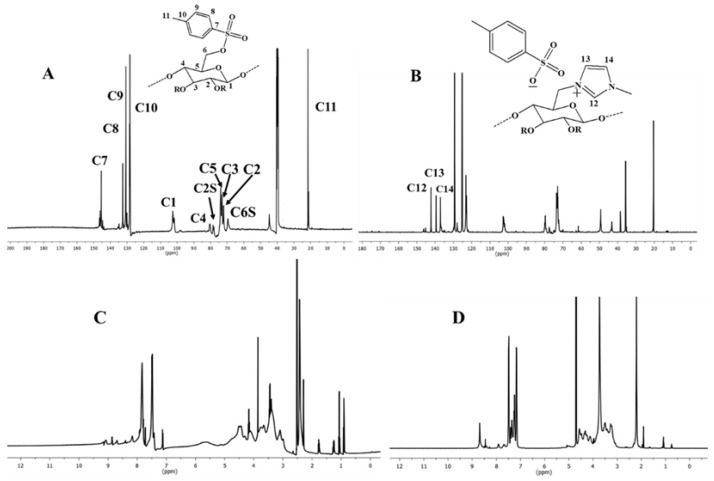
^13^C NMR and ^1^HNMR spectroscopy for tosyl cellulose (**A**,**C**) and CIMD (**B**,**D**).

A water-soluble cellulose derivative containing imidazolium tosylate was synthesized via a nucleophilic substitution reaction. The replacement of the tosyl groups with imidazolium was proven using analytical procedures including ^13^C NMR and ^1^H NMR. Figure 1 illustrates the spectrum of cationic cellulose obtained with NMR spectroscopy.

The aromatic carbon signals of the tosyl cellulose range from 128 to 145 ppm. The glucose unit C1 is represented by the signal at 103 ppm. The signals for the glucose carbon centers range between 81 and 73 ppm. The tosylated C6 may be responsible for the signal at 68.8 ppm, while the non-tosylated C6 may be responsible for the signal at 61 ppm. The peak at 21 ppm may refer to the C11 in the tosyl group. However, the cationic cellulose exhibits a new peak at 36 ppm, which may be assigned to the formation of N-CH_3_. Moreover, new signals are produced at 137, 140, and 142 ppm by carbons in the imidazolium group. According to the ^13^C NMR spectroscopy described above, imidazolium tosylate was formed on the cellulose. 

The ^1^H NMR spectra of the tosyl cellulose and CIMD are shown in Figure 1C,D. The anhydroglucose units show proton resonances at 3.1–4.7 ppm in the ^1^H NMR spectrum of the tosyl cellulose. The peak at 7.4–7.9 ppm may be assigned to the phenyl protons of the tosyl group. Moreover, the signal at 2.4 confirms the protons from the CH_3_ of the tosyl group. New signals are observed at 7.3, 7.4, and 8.7, which prove the incorporation of the methyl imidazolium group. This finding is also confirmed by the measurements of the Z potential, as shown in Table 1. The presence of cationic species like CIMD is confirmed by the positive value of the Z potential, in contrast to the negative ones measured in the case of the control (i.e., PCL nanofibers). Similar values were obtained in the case of the alginate coating, which was able to neutralize the positive charges on the fibers’ surface due to the presence of the CIMD.

### 2.2. Morphology and Microstructure

**Figure 2:**
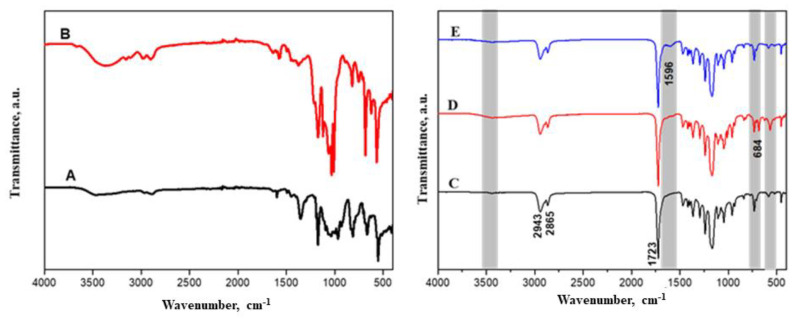
FTIR spectroscopy of tosyl cellulose (**A**), CIMD (**B**), PCL (**C**), PCL/CIMD (**D**), and PCL/CIMD/SA (**E**).

ATR-FTIR spectroscopy was utilized to distinguish the PCL-based samples after mixing with the CIMD and the sodium alginate. Figure 2A shows the characteristic vibration bands of the CIMD. The weak signal at 3341 cm^−1^ describes the hydroxyl groups and the inter- and intramolecular hydrogen bonds. Furthermore, the signal at 2887 cm^−1^ relates to the C-H stretching of the methyl groups. The characteristic signals of the tosyl groups at 1163 cm^−1^ (υs SO_2_), 1359 cm^−1^ (υas SO_2_), and 1540 cm^−1^ (ν C=C aromatic) confirm their presence as counter anions [24]. Moreover, the decrease in the signal at 3405 cm^−1^ suggests a low density of the hydroxyl groups. The peak around 1559 cm^−1^ becomes stronger due to the additional C-N stretch characteristic in the methyl-imidazolium.

The main adsorption peaks for the PCL were assigned at 2900 cm^−1^, which was assigned to the -CH_2_ stretching vibrations, and at 1722, which was assigned to ν(C=O). Moreover, bending modes (δ) of the -CH_2_ groups were revealed at 1469 cm^−1^, and, for ν (C-O), they were noticed at 1173 cm^−1^ [25]. After immersing the PCL in the alginate solution to form a polyelectrolyte, the main adsorption peaks of the alginate were assigned. The broad band measured around 3450 cm^−1^ was assigned to the stretching vibrations (ν) of the hydroxyl groups. Moreover, the asymmetric (ν_a_) and symmetric (ν_s_) stretching of the carboxylic groups were located at 1600 cm^−1^ and at 1415 cm^−1^.

**Figure 3:**
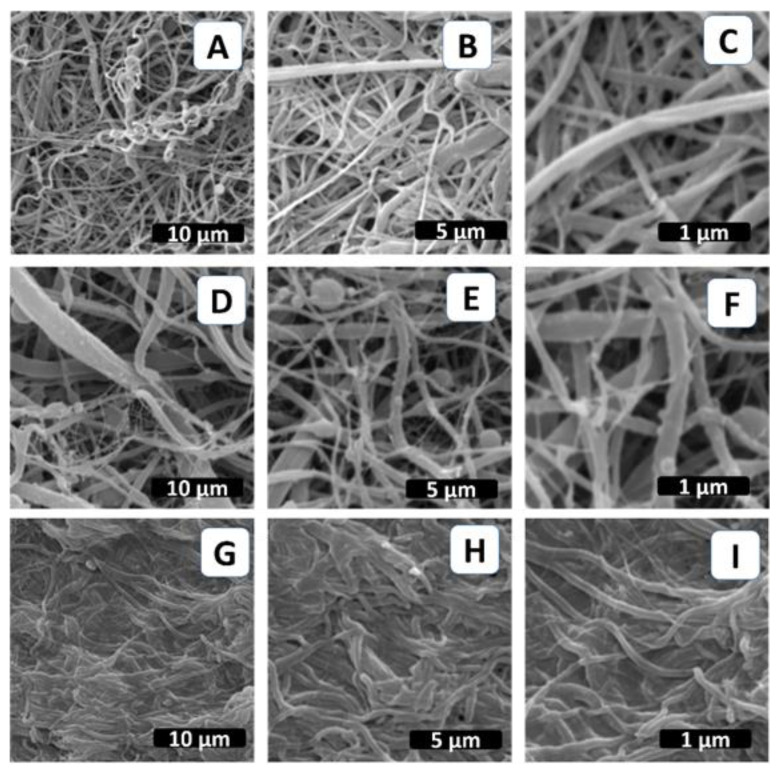
SEM images of the fabricated PCL mats: (**A**–**C**) PCL nanofibers, (**D**–**F**) PCL/CIMD nanofibers, and (**G**–**I**) PCLCIMD/SA polyelectrolyte complex.

Although electrospinning is affected by various processing parameters, the solvent system plays a crucial role in the formation of blend nanofibers from polymer blends and composites. PCL nanofibers have been produced successfully using different organic solvents such as acetone, chloroform, dichloromethane, and tetrafluoroethylene [26,27]. In this study, a formic acid/acetic acid (FA/AA) system was used as a common solvent of polycaprolactone and CIMD. The SEM micrographs of the pure PCL and PCL/CIMD fibers produced using the formic acid/acetic acid (FA/AA) are given in Figure 3. It can be observed that the fiber diameter of the PCL sample is in the range of 571 ± 206 nm, which is less than the PCL/CIMD sample (834 ± 378 nm). Figure 3 also shows the morphology of the PCL/CIMD mats after the treatment with alginate to form the polyelectrolyte complex’s layer. The formation of a smooth layer on the surface of the PCL/CIMD electrospun fiber indicates the ability of an electrospun PCL/CIMD fiber (as a cationic electrolyte) and alginate (as an ionic electrolyte) to form polyionic-complexed mats.

**Figure 4:**
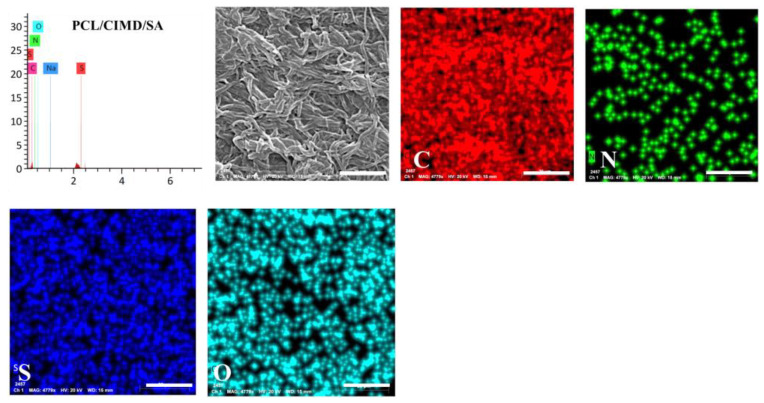
EDX mapping for the PCL/CIMD/SA nanofibers.

We also carried out an energy dispersive X-ray spectroscopy (EDX) mapping for the PCL/CIMD/SA sample, and the results are shown in Figure 4. The PCL/CIMD/SA nanofibers show a uniform distribution of C, O, N, and S over the surface of the fibers. This indicates a homogenous mixing of the cationic cellulose with the PCL during the electrospinning process.

### 2.3. Water Uptake and Degradation

**Figure 5:**
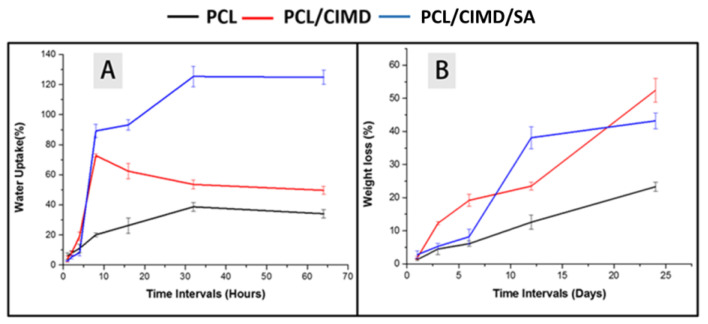
(**A**) water uptake and (**B**) degradation/weight loss of the PCL, PCL/CIMD mats, and PCL/CIMD/SA membrane samples, showing the changes in sample weight after immersion in PBS.

Figure 5 A,B show the water uptake of the prepared electrospun mats and the weight loss after their immersion in water for 1, 2, 4, 8, 16, 32, and 64 h. It was observed that the water uptake ratio of the PCL, PCL/CIMD, and PCL/CIMD/SA samples was 20.1%, 62.8%, and 89.2% in the first 8 h, respectively. However, after 32 h, the water uptake ratio of both the PCL mat and the PCL/CIMD/SA membrane increased to 38.7% and 125.4%, respectively, and decreased to 51% for the PCL/CIMD mats. In general, the results showed that the water absorption ratio of the polyelectrolyte membranes was significantly higher than that in the PCL and PCL/CIMD mats. In addition, the water uptake of the PCL/CIMD mats decreasing with time, which could have been due to the release of the CIMD.

In the past decade, degradable biomaterials of natural or synthetic origin have been introduced as the preferred candidates for developing scaffolding materials for tissue engineering and regenerative medicine applications. The in vitro weight loss is a well-known measurement for probing the biomaterial degradation behavior of a given material under a physiological environment. According to the degradation tests (Figure 5B), the weight loss among the samples was not significant, reaching about 2% after 1 day. After that, the degradation became faster for the PCL/CIMD sample, reaching 12.3% of weight loss after 3 days. On the other hand, the weight loss rate for both the PCL sample and the PCL/CIMD/SA membrane showed a slow degradation potential until day 6, reaching 8.2% of weight loss. Finally, it was found that the weight loss ratios of the PCL/CIMD and PCL/CIMD/SA samples increased with the incubation time and that the weight loss of the PCL mats was ~23% at the end of the 24 days, which was obviously lower than the PCL/CIMD mat’s and the PCL/CIMD/SA membrane’s weight loss of ~52.8 and 43.2%.

### 2.4. Biocompatibility

**Figure 6:**
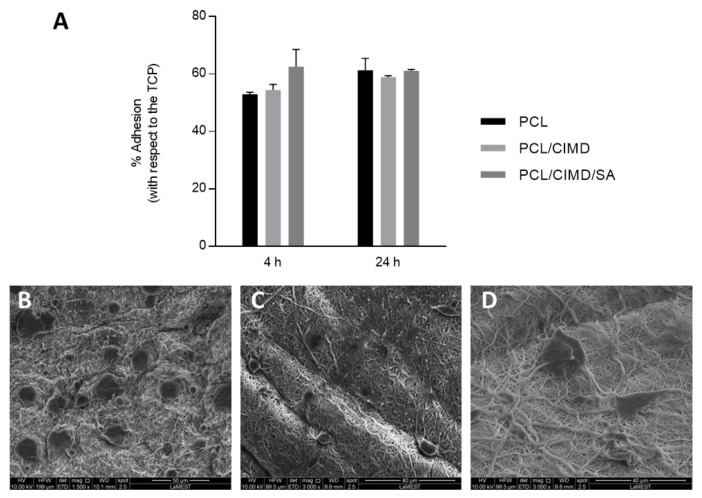
(**A**) Cell adhesion assay. The results are presented as the percentage of cell adhesion with respect to the control (TCP). SEM images of seeded hMSCs after 24 h onto the PCL (**B**), PCL/CIMD (**C**), and PCL/CIMD/SA (**D**) samples.

To evaluate the effect of the cationic cellulose derivative on the cells, adhesion and proliferation assays were performed with human mesenchymal stem cells (hMSCs) (Figure 6). After 4 and 24 h of cell culture, no significant differences were detected between the groups independently of their composition. However, after 4 h, the PCL/CIMD/SA fibers showed a better cell adhesion, which could be related to the improvement of its hydrophilicity brought about by the presence of the alginate with respect to the PCL/CIMD fibers, while, after 24 h, the cell adhesion was very similar between the groups, with a slight increase with respect to the 4 h assay in the PCL and PCL/CIMD fibers. From the SEM images, it is possible to evaluate the morphology of the cells in contact with the fibers after 24 h. In the case of the PCL fibers, the density of the cells is greater with respect to the other materials; however, the morphology of the cells is preferentially rounded. In the presence of cationic cellulose (PCL/CIMD), the cells are more spread out, which could be related to the positive-charged surface facilitating cell adhesion and increasing cell spreading. Similarly, the cells seeded onto the PCL/CIMD/SA fibers tend to well spread along the fibers, also because of the hydrophilic properties of this surface. 

**Figure 7:**
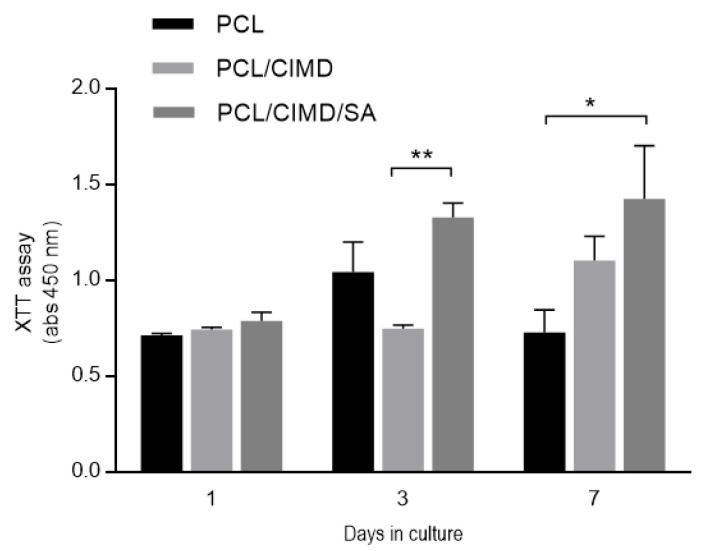
Cell viability of the hMSCs seeded onto the PCL, PCL/CIMD, and PCL/CIMD/SA samples. The graph represents the mean ± standard error deviation. Significant differences are presented as * *p* < 0.05 and ** *p* < 0.01.

The proliferation of the hMSCs was evaluated after 1, 3 and 7 days on all the sample typologies. The results showed a significant increase in the metabolic activity of the cells just after 3 days, in the presence of the CIMD/SA complex, with respect to the controls (Figure 7). After 7 days, the proliferation of the hMSCs seeded onto the PCL/CIMD and PCL/CIMD/SA samples further increased, differently to the PCL fibers, which showed a stop in their cell growth, thus confirming the beneficial effect of CIMD/SA functionalization on hMSCs’ response.

### 2.5. Antimicrobial Study

**Figure 8:**
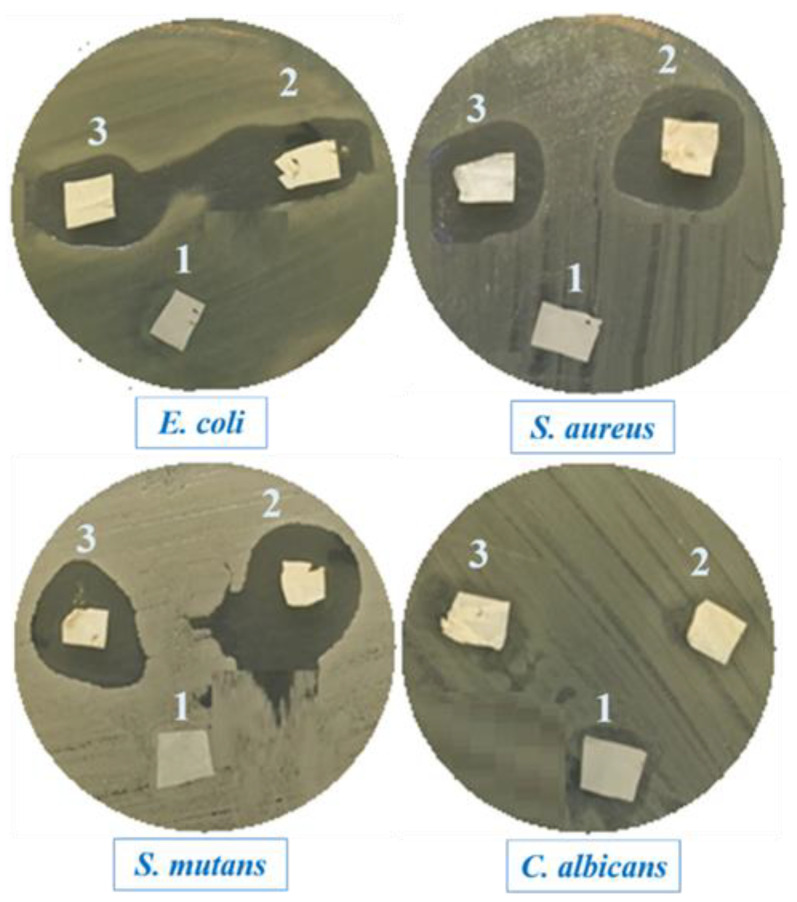
Antimicrobial activity expressed as the halo-zones of the PCL, PCL/CIMD, and PCL/CIMD/SA mats, coded as one, two, and three, respectively, against four pathogenic microbes.

The antimicrobial activities of the PCL, PCL/CIMD, and PCL/CIMD/SA electrospun mats were explored on different pathogenic strains and investigated using the disc diffusion technique. The obtained data are represented in Table 2 and Figure 8. The PCL/CIMD mat showed a higher antimicrobial activity than the other prepared composite film incorporated with SA; these results indicate that the activity of the SA during the antimicrobial evaluation was not significant and had no positive effects against all the pathogenic microbes used. The data obtained from both the PCL/CIMD and PCL/CIMD/SA mats showed a higher antimicrobial activity against *S. mutans*, followed by *S. aureus*, and, then, *E. coli*, while *C. albicans* exhibited more resistance against all the prepared composite films. From our data, only the effect of the PCL mat against all the pathogenic microbes used was negligible; this is in agreement with other reported studies [28]. We can conclude that antimicrobial activity can be strictly associated with the presence of CIMD, in agreement with previous experimental evidence [29,30]. The mechanism of action of the CIMD against pathogenic microbes is related to its alkyl chain, which contains more than ten carbon atoms—for example C12 and C14—and the activity of CIMD is dependent on the length of said alkyl chain. Other mechanisms are related to the presence of the hydroxyethyl chain and methyl groups in the CIMD structure [31].

## 3. Materials and Methods

### 3.1. Materials

Microcrystalline cellulose, p-toluenesulfonyl chloride, sodium alginate (SA), and 1-methylimidazole were purchased from Sigma Aldrich (Milan, Italy). Also, 1-Butyl-3-methylimidazolium chloride was bought from *IoLiTec* (Heilbronn, Germany). Polycaprolactone (Mw 70,000–90,000, Sigma-Aldrich), formic acid (85%, Edwic, Cairo, Egypt), and acetic acid (96%, Edwic, Egypt) were used as purchased, without any purification. In addition to these chemicals, the rest were of analytical grade and did not require further purification.

### 3.2. Preparation of the Cellulose Containing Imidazolium Tosyalate

Cellulose (particle size distribution 18–22 µm, DP ~1000, degree of crystallinity 91%) containing imidazolium tosyalate was prepared according to our previous work [32,33]. In a round-bottom flask, 1 g of tosyl cellulose was dissolved in DMSO. After its complete dissolution, 3.1 g of 1-methyl imidazole was added under continuous stirring at 100 °C. After 12 h, the reaction mixture was poured into 100 mL of ethyl acetate. The separated product was washed with ethyl acetate several times and then dried in a vacuum at 50 °C. The amount of imidazolium-tosylate-containing cellulose produced was around 3.2 g. Its elemental composition was as follows: C 4.8 ± 0.3%, H 4.5 ± 0.2%, N 5.5 ± 0.4%, and S 4.9 ± 0.2%.

### 3.3. Electrospun Fibers

The PCL solution (15 wt%) was prepared in a formic acid/acetic acid solvent system (70:30 *v*/*v*) and left under magnetic stirring for 2 h until its complete dissolution. Then, cell-imidazolium powder was mixed with the PCL solution in different proportions (PCL/CIMD = 100:0, 90:10, and 85:15) to yield a 10 wt% solution. The prepared PCL and PCL-CIMD solutions were then injected into a 3 mL syringe with a 19G needle under 17 kV of applied voltage and at a distance of 15 cm from the needle’s tip to the collector. The voltage was adjusted using a Glassman high voltage series covering a range of 0–20 kV. A syringe pump series 100 regulated the flow rate of the solution. The electrospinning was performed at room temperature (about 22 °C) and at a relative humidity (RH) of 60 ± 2%.

### 3.4. Polyelectrolyte Complex Deposition

The electrospun PCL/CIMD nanofibers were coated with SA as an anionic macromolecule; in brief, SA was dissolved for 4 h in water at a concentration of 0.2% (*w*/*v*). The obtained PCL/CIMD mats were immersed in 50 mL of the SA solution for 10 min. After that, the PCL/CIMD mats were rinsed twice in 100 mL of to remove the SA in excess from the surface. Finally, the fiber mats were dried at 40 °C in a vacuum oven for 24 h. The coated nanofibers were kept under vacuum before further characterization.

### 3.5. Microstructure Analysis

The morphology and fiber diameter were observed using a field-emission scanning electron microscope (SEM, Jeol JXA 840, Tokyo, Japan). The mean fiber diameter of the samples was determined by measuring about 50 individual fibers using an image analysis software (Image J 1.42q, NIH, Bethesda, MA, USA). The Fourier-transform infrared (FTIR) spectroscopy was performed with an attenuated total reflectance-FTIR spectroscope/Varian IR spectrometer (Agilent, Santa Clara, CA, USA). The Z potential of the fibers’ surface was investigated via dynamic light scattering (DLS) (Malvern Zetasizer NanoZS, Worcestershire, UK). The nanofibers were dispersed in 1 mL of water and analyzed with a zeta dip cell.

### 3.6. Degradation and Water Uptake

The obtained mats were designed to be 1 × 1 cm^2^ and weighed; this measurement was labelled as the initial weight (W_i_) of the mats. Afterwards, the samples were immersed into 15 mL of phosphate buffer solution (PBS) at 37 °C for different time intervals. After each specific period, the mats were dried using a filter paper in order to remove the surface solution; then, the samples were weighed; this measurement was indicated as the wet weight (W_w_). The samples were weighed again after drying for 24 h, and this was termed as the dry weight (W_d_). Following Equations (1) and (2), the weight loss percentage and water uptake percentage were calculated:

(1) $$\mathrm{Weight}\;\mathrm{loss}\;(\%)=\frac{W_{w}{-W}_{i}\;}{W_{i}}x\;100$$

(2) $$\mathrm{Water}\;\mathrm{uptake}\;(\%)=\frac{W_{w}{-W}_{i}\;}{W_{i}}x\;100$$

### 3.7. Biocompatibility Assays

#### 3.7.1. Cell Culture

hMSCs (SCC034), fetal bovine serum (FBS), Cell Proliferation Kit II (XTT, Roche Diagnostics Deutschland GmbH, Mannheim, Germany), phosphate buffer solution (PBS), streptomycin, penicillin, and L-glutamine were purchased from Sigma Aldrich, Milan, Italy.

The hMSCs (from four–five passages) were cultured until 80% of confluence, in a 75 cm^2^ cell culture flask, in Eagle’s alpha minimum essential medium (α-MEM) supplemented with 10% of FBS, antibiotic solution (streptomycin 100 µg/mL and penicillin 100 U/mL), and 2 mM of L-glutamine, at 37 °C, in a humidified atmosphere with 5% of CO_2_ and 95% of air. For the in vitro studies, the electrospun fibers were cut and placed in a 96-cell culture plate. Then, the samples were sterilized in an ethanol solution (70%) for 30 min. Afterwards, the samples were washed three times with PBS and dried under the hood.

#### 3.7.2. In Vitro Assays

For adhesion and proliferation, a Cell Proliferation Kit II (XTT, Roche Diagnostics Deutschland GmbH, Mannheim, Germany) was purchased (Sigma-Aldrich, Milan, Italy). For cell adhesion, 1 × 10^4^ hMSCs were seeded onto the PCL, PCL/CIMD, and PCL/CIMD/SA mats in cell culture standard conditions for 4 and 24 h. After this period, the medium was removed, and the samples were washed with PBS to remove the unattached cells. Cell culture medium with an XTT working solution was added to incubate the samples for four hours. The supernatant was recovered after that time and placed in a 96-well plate reader to record the absorbance measurements at 450 nm (Wallac Victor 1420, PerkinElmer, Boston, MA, USA). The results were presented as the percentage of cell adhesion with respect to the tissue culture plate (TCP).

After 24 h in the cell culture, the cell morphology of the hMsCs was examined using scanning electron microscopy (SEM, QuantaFEG 200, FEI, Eindhoven, The Netherlands). The scaffolds were washed three times with PBS and fixed with 4% of formaldehyde. They were dehydrated using a graded series of ethanol (25–100%) solutions and, then, air-dried. Representative images were taken to observe the morphology and spread of the cells onto the nanofibers.

Cell proliferation was performed with an XTT assay kit. Briefly, after 1, 3, and 7 days, the cell culture medium was removed and changed for the cell culture medium with the XTT working solution as indicated in the manufacturer’s instructions. After 4 h of incubation, the supernatant was collected to measure the absorbance at 450 nm. The results were presented as mean ± standard error deviation (n = three). An Analysis of variance (ANOVA) with Tukey’s post hoc test was used to detect differences between the groups. A value of *p* < 0.05 was considered to determine statistically significant differences.

### 3.8. Antimicrobial Studies

#### 3.8.1. Microorganisms and Growth Conditions

The antimicrobial activity of the newly synthesized complexes was assessed against four selected pathogens, including Gram-negative bacteria Escherichia coli ATCC 25922 (*E. coli*), Gram-positive bacteria Staphylococcus aureus ATCC 25923 (*S. aureus*), Streptococcus mutant ATCC 25175 (*S. mutans*), and yeast Candida albicans ATCC 10231 (*C. albicans*). All of the pathogenic microbes were obtained from the American Type Culture Collection (ATCC). The microbial strains were cultivated in a Mueller Hinton broth medium composed of 0.15% (*w*/*v*) of starch, 1.75% (*w*/*v*) of acid hydrolysate of casein, and 0.2% (*w*/*v*) of beef extract incubated at 30 °C under shaking at 200 rpm for one day.

#### 3.8.2. Disc Diffusion Technique

The antimicrobial assessments of the PCL, PCL/CIMD, and PCL/CIMD/SA mats were qualitatively examined using the disc diffusion technique, according to our previous study [5] with little change. All the films were cut into 0.5 cm^2^ and sterilized for 30 min under ultra violet (UV) irradiation before application to ensure aseptic conditions. At the beginning, about 15 mL of sterilized Mueller Hinton medium (20 g/L agar) was poured on the Petri dish until it solidified. The pathogenic suspension (10^8^ CFU/mL) with an approximately equal density to the 0.5 McFarland standards was evenly spread over the Petri dish. Afterward, the tested films were placed on the inoculated agar plates. The plates were placed in the refrigerator at 4 °C for 1 h to allow the tested sample to diffuse, temporarily inhibiting the model microbes before being cultivated at 37 °C for one day. After the cultivation time, the plates were evaluated for antimicrobial properties by measuring the diameter of the inhibition zone (including the film disc). The values were averaged from three independent experiments.

## 4. Conclusions

The preparation of a polyelectrolyte-based membrane made on PCL electrospun fibers functionalized with CIMD and SA was proposed. The CIMD was synthesized and incorporated into the PCL nanofibers during the electrospinning process, while the SA was integrated via self-assembly only after the fabrication of the membrane as a fiber coating. The polyelectrolyte interactions between the CIMD and SA were widely investigated in order to establish a correlation among the main fiber properties in terms of morphology, surface charge, water adsorption, and in vitro degradability. It was demonstrated that the addition of SA does not compromise the antimicrobial properties imparted by the CIMD against different populations of pathogenic microbes (i.e., *S. mutans*, *S. aureus*, *E. coli)*, but that it supports the in vitro response of cells in terms of their viability up until 7 days. In this view, the functionalization of PCL nanofibers via CIMD/SA concretely suggests a promising strategy to be used in the fabrication of more efficient membranes for wound healing applications.

## Figures and Tables

**Table 1:** ζ-potential measurements.

Sample	Ζ-Potential [mV]
PCL	−19.1 ± 1.24
PCL/CIMD	7.85 ± 2.22
PCL/CIMD/SA	−15.2 ± 5.03

**Table 2:** Antimicrobial activity of the PCL, PCL/CIMD, and PCL/CIMD/SA mats against four pathogenic microbes.

Pathogenic Microbes	Diameters of the Inhibition Zone (mm)		
	PCL	PCL/CIMD	PCL/CIMD/SA
*E. coli*	0.0 ± 0.0	19 ± 1.17	17 ± 1.09
*S. aureus*	0.0 ± 0.0	20 ± 1.21	18 ± 1.61
*S. mutans*	0.0 ± 0.0	22 ± 1.19	19 ± 1.51
*C. albicans*	0.0 ± 0.0	7 ± 0.06	6 ± 0.04

## Data Availability

Not available.

## Sample Availability

Not available.
